# Hybridization and introgression in sympatric and allopatric populations of four oak species

**DOI:** 10.1186/s12870-021-03007-4

**Published:** 2021-06-09

**Authors:** Xuan Li, Gaoming Wei, Yousry A. El-Kassaby, Yanming Fang

**Affiliations:** 1 Co-Innovation Center for Sustainable Forestry in Southern China, College of Biology and the Environment, Key Laboratory of State Forestry and Grassland Administration On Subtropical Forest Biodiversity Conservation, Nanjing Forestry University, 159 Longpan Road, Nanjing, 210037 PR China; 2 Department of Forest and Conservation Sciences Faculty of Forestry, The University of British Columbia, 2424 Main Mall, Vancouver, BC V6T 1Z4 Canada; 3 School of Physics and Electronics Henan University, Jinming Avenue, Jinming District, Kaifeng, 475001 PR China

**Keywords:** NSSR, Section *Quercus*, Section *Cerris*, Hybridization, Introgression

## Abstract

**Background:**

Hybridization and introgression are vital sources of novel genetic variation driving diversification during reticulated evolution. *Quercus* is an important model clade, having extraordinary diverse and abundant members in the Northern hemisphere, that are used to studying the introgression of species boundaries and adaptive processes. China is the second-largest distribution center of *Quercus*, but there are limited studies on introgressive hybridization.

**Results:**

Here, we screened 17 co-dominant nuclear microsatellite markers to investigate the hybridization and introgression of four oaks (*Quercus acutissima*, *Quercus variabilis*, *Quercus fabri*, and *Quercus serrata*) in 10 populations. We identified 361 alleles in the four-oak species across 17 loci, and all loci were characterized by high genetic variability (*H*_E_ = 0.844–0.944) and moderate differentiation (*F*_ST_ = 0.037–0.156) levels. A population differentiation analysis revealed the following: allopatric homologous (*F*_ST_ = 0.064) < sympatric heterogeneous (*F*_ST_ = 0.071) < allopatric heterogeneous (*F*_ST_ = 0.084). A Bayesian admixture analysis determined four types of hybrids (*Q. acutissima* × *Q. variabilis*, *Q. fabri* × *Q. serrata*, *Q. acutissima* × *Q. fabri*, and *Q. acutissima* × *Q. variabilis* × *Q. fabri*) and their asymmetric introgression. Our results revealed that interspecific hybridization is commonly observed within the section *Quercus*, with members having tendency to hybridize.

**Conclusions:**

Our study determined the basic hybridization and introgression states among the studied four oak species and extended our understanding of the evolutionary role of hybridization. The results provide useful theoretical data for formulating conservation strategies.

**Supplementary Information:**

The online version contains supplementary material available at 10.1186/s12870-021-03007-4.

## Background

Introgressive hybridization is a common natural phenomenon among biological organisms, especially oaks [1]. Interspecific hybridization is an important source of genetic variation and drives diversification in response to changing environmental conditions [2–4]. Additionally, introgression may have ecological consequences, including shaping community assembly and structure [5, 6]. Interspecific introgression of adaptive genetic variation has been documented among forest tree species, including poplars [7], eucalypts [8], spruces [9], pines [10], and oaks [11]. Nuclear DNA markers, such as microsatellites (SSRs) [12], randomly amplified polymorphic DNA markers [13], and chloroplast DNA [14] have been used to detect introgression across species. With advancements in genomic studies, our understanding of the ecological, genetic, and genomic factors underlying the diverse outcomes of interspecific introgression in hybrid zones is increasing [15–17].

The genus *Quercus* L. (oaks) contains more than 400 species that are widespread in the Northern hemisphere [18]. Oak is an important model clade that offers fundamental insights into the ecological and evolutionary consequences of hybridization and introgression [19]. In *Quercus*, naturally occurring interspecific hybrids are common [11, 15]. Within *Quercus*, the American oaks and European white oaks have been well-studied [20]. For example, high quality SNPs from genic regions have been used to characterize the introgression patterns among European white oaks (*Quercus petraea* and *Quercus robur*) [21]. Eaton et al. (2015) utilized genomic RAD-seq data sampled from American live oaks (*Quercus* series *Virentes*) for phylogenetic inference and determining introgressions between lineages [22]. In China, most oak hybridization studies have used nuclear DNA markers, such as SSRs and AFLPs [23]. For conservation purposes, it is necessary to study the hybridization and introgression of oak, especially native species.

China is the second-largest distribution center of *Quercus*, with 62 species described in the *Flora of China* that are divided into five morphology-based sections: *Quercus*, *Aegilops*, *Heterobalanus*, *Engleriana*, and *Echinolepides* [24–26]. The first two sections (*Quercus* and *Aegilops*) are composed of deciduous oak species which correspond to the *Quercus* and *Cerris* proposed by Denk [27]. For our present study, *Quercus acutissima* Carruthers and *Quercus variabilis* Blume belong to Group *Cerris*, while *Quercus fabri* Hance and *Quercus serrata* Murray belong to Group *Quercus*. A new classification of *Quercus* L. was proposed by Denk harbouring eight sections: *Cyclobalanopsis*, *Cerris*, *Ilex, Lobatae*, *Quercus*, *Ponticae*, *Protobalanus*, and *Virentes* [27]. The phylogenetic positions of our study species did not change, as they remain in *Cerris* and *Quercus* Groups*.*

The studied four oak species (*Q. acutissima*, *Q. variabilis*, *Q. fabri*, and *Q. serrata*), having an overlapping distribution in subtropical areas, are important species in subtropical mountain and warm temperate deciduous broad-leaved forests, which occupy important positions in China’s forest ecosystem [28]. In our study, we aimed to: (1) identify the pattern of hybridization between these four oak species; (2) investigate whether, and to what extent, introgression exists among these four-oak species; and (3) compare the introgression intensity levels in sympatric and allopatric populations. This work provides an understanding of the evolutionary mechanisms of *Quercus*, as well as the biological processes behind their biodiversity, and has implications for forest management.

## Results

### The availability of microsatellite loci (SSRs)

The average values of the estimated null allele frequency at each locus across all populations were all less than 0.2 (Table 1). No evidence of significant linkage disequilibrium was observed for each pair of loci in each population at the indicative adjusted nominal level (5%) for multiple comparisons equal to 0.000368 (Table S1). The 17 polymorphic SSRs generated 361 alleles, with a mean of 21 alleles per locus. Some SSRs were highly variable, including quru-GA-0M05, Quru-GA-1H14, Quru-GA-1i15, MSQ16, ssrQpZAG36, and ssrQrZAG112, which each contained more than 21 alleles. Mean *PIC*, *A*_R_, and *H*_S_ for all markers were 0.909 (range: 0.844 to 0.939), 10.7 (range:7.1 to 14.2), and 0.550 (range: 0.254 to 0.980), respectively (Table 1).

**Table 1:** Genetic statistics of 17 nuclear microsatellite loci used in this study

	Null	*N*_A_	*N*_E_	*H*_O_	*H*_E_	*A*_R_	*H*_S_	*PIC*	*F*_ST_	c*F*_ST_	*G'*_ST_	*N*_*m*_
QM58TGT	0.011	21	17.2	0.980	0.944	9.9	0.980	0.939	0.095*	0.096	0.088*	2.24
quru-GA-0I01	0.097	20	10.3	0.390	0.904	9.7	0.383	0.895	0.081*	0.053	0.074*	2.35
quru-GA-0M05	0.078	26	16.8	0.450	0.942	12.9	0.452	0.937	0.046*	0.043	0.042*	3.64
quru-GA-0M07	0.073	16	10.8	0.420	0.909	10.1	0.416	0.900	0.076*	0.061	0.070*	2.49
Quru-GA-Oi21	0.109	18	7.6	0.267	0.870	8.7	0.254	0.857	0.161*	0.132	0.151*	1.19
Quru-GA-1H14	0.019	29	15.9	0.797	0.939	14.2	0.794	0.934	0.047*	0.048	0.042*	4.01
Quru-GA-1i15	0.076	25	9.3	0.483	0.894	11.0	0.487	0.885	0.084*	0.062	0.079*	2.27
MSQ16	0.045	29	13.9	0.567	0.930	13.7	0.571	0.924	0.047*	0.038	0.042*	3.80
ssrQpZAG1/5	0.086	21	13.7	0.393	0.929	11.9	0.389	0.922	0.038*	0.033	0.033*	4.20
ssrQpZAG15	0.054	21	12.0	0.483	0.918	10.4	0.485	0.911	0.112*	0.086	0.098*	1.87
ssrQpZAG36	0.027	25	14.6	0.673	0.933	11.3	0.665	0.928	0.123*	0.096	0.111*	1.72
ssrQrZAG 7	0.036	19	12.3	0.697	0.920	13.2	0.698	0.920	0.037*	0.033	0.034*	4.60
ssrQrZAG 31	0.047	21	11.6	0.560	0.916	11.7	0.563	0.916	0.128*	0.102	0.114*	1.64
ssrQrZAG 74	0.057	16	6.4	0.360	0.844	7.1	0.358	0.844	0.204*	0.168	0.190*	0.95
ssrQrZAG 87	0.051	12	9.2	0.667	0.893	7.6	0.663	0.892	0.152*	0.141	0.150*	1.27
ssrQrZAG 96	0.091	20	11.2	0.330	0.913	8.5	0.324	0.912	0.156*	0.105	0.142*	1.29
ssrQrZAG112	0.025	22	14.7	0.870	0.933	9.7	0.872	0.933	0.148*	0.144	0.130*	1.50
overall	0.058	21	12.2	0.552	0.914	10.7	0.550	0.909	0.099	0.085	0.093*	2.41

*N*_A_ number of alleles, *N*_E_ effective number of alleles, *A*_R_ allelic richness with rarefaction to the common sample size of 10, *H*_O_ observed heterozygosity averaged across loci, *H*_E_ expected heterozygosity averaged across loci, *H*_S_ genetic diversity within populations averaged across loci, *PIC* polymorphism information content, *F*_ST_ genetic differentiation index (Weir and Cockerham 1984), *cF*_ST_ corrected genetic differentiation index using the “exclusion null alleles” (ENA) method (Chapuis & Estoup 2007), *G'*_ST_ standardized genetic differentiation index (Hedrick 2005), *T*_A_ annealing temperature, *null* null allele frequency averaged across populations; *indicates statistical significance (*P* < 0.001) based on 10,000 permutations implemented in ARLEQUIN

Comparison of DIC values across the 10 populations showed that models with null alleles had higher support than models without null alleles, and the average estimated frequency of across loci was slightly high (ranging from 0.102 to 0.188) (Table 2; Table S1). The inbreeding coefficient of the 10 populations was large (mean = 0.41) and significantly deviated from zero (*P* < 0.05). Two populations, LT-F and ZW-B, did not departure from Hardy–Weinberg equilibrium, and seven populations, BY-A, BY-V, LT-B, ZW-V, ZJ-A, ZJ-V, ZJ-F, and ZJ-B, did not departure from Hardy–Weinberg equilibrium after excluding the null alleles bias.

**Table 2:** Genetic statistics of the studied 10 oak populations

Population	Null	*N*_A_	*N*_E_	*H*_O_	*H*_E_	*A*_R_	*H*_S_	*F*_IS_	*F*_IS_'
BY-A	0.140^a^	12.8	7.9	0.579	0.844	11.8	0.848	0.317*	0.027
BY-V	0.170^a^	10.2	6.3	0.505	0.791	9.6	0.797	0.366*	0.008
LT-F	0.102^a^	12.6	7.1	0.692	0.854	11.1	0.856	0.192*	0.005^a^
LT-B	0.110^a^	11.3	6.6	0.610	0.821	11.2	0.831	0.267*	0.042
ZW-V	0.181^a^	9.3	5.8	0.465	0.777	8.7	0.782	0.406*	0.018
ZW-B	0.169^a^	11.1	6.1	0.516	0.817	10.2	0.822	0.373*	0.021^a^
ZJ-A	0.106^a^	11.6	6.6	0.492	0.828	10.6	0.834	0.410	0.186
ZJ-V	0.188^a^	11.6	6.3	0.443	0.818	10.8	0.824	0.463*	0.065
ZJ-F	0.137^a^	12.6	7.4	0.579	0.858	11.5	0.862	0.328*	0.035
ZJ-B	0.111^a^	11.6	6.7	0.621	0.826	11.0	0.830	0.252*	0.023
mean		11.5	6.7	0.550	0.753	10.7	0.829		

*N*_A_ number of alleles, *N*_E_ effective number of alleles, *A*_R_ allelic richness with rarefaction to the common sample size of 10, *H*_O_ observed heterozygosity averaged across loci, *H*_E_ expected heterozygosity averaged across loci, *H*_S_ genetic diversity within populations averaged across loci, *F*_IS_ inbreeding coefficient, *F*_IS_*'* corrected inbreeding coefficient estimated in INEST version 2.2 (Chybicki and Burczyk 2009) based on the *nfb* model (where n = null alleles, f = inbreeding coefficient and b = genotyping failures). ^a^Deviance information criterion (DIC) values support the significance of null alleles or inbreeding in the *nfb* model; *P < 0.05

### Analyses of genetic diversity among the four oaks

At the DNA level, the four oak species had high degree of genetic diversity where mean *N*_E,_*A*_R_, and *H*_S_ were 6.7 (range: 5.8 to 7.9), 10.7 (range: 8.7 to 11.8), and 0.829 (range: 0.782 to 0.862), respectively (Table 2). The four species were divided into two groups belonging to *Cerris* and *Quercus* (groups AV and FB) based on the STRUCTURE analysis. The *H*_o_ of group FB (0.607) was greater than that of group AV (0.497) as determined using FSTAT (*P* < 0.05). The highest intraspecific genetic diversity (*A*_R_, *H*_S_, and *H*_o_) was observed in the *Q. fabri* population compared with the three other investigated species and was significantly different from *Q. variabilis* (*P* < 0.05). Among the four different sites, Liangting had highest *H*_o_, which was significantly different from that of Zhongwu. Significant differences in *A*_R_ were observed between Zijin and Zhongwu while *H*_S_ and *F*_ST_ showed no significant differences among sites.

### Genetic differentiation and population structure

**Fig. 1:**
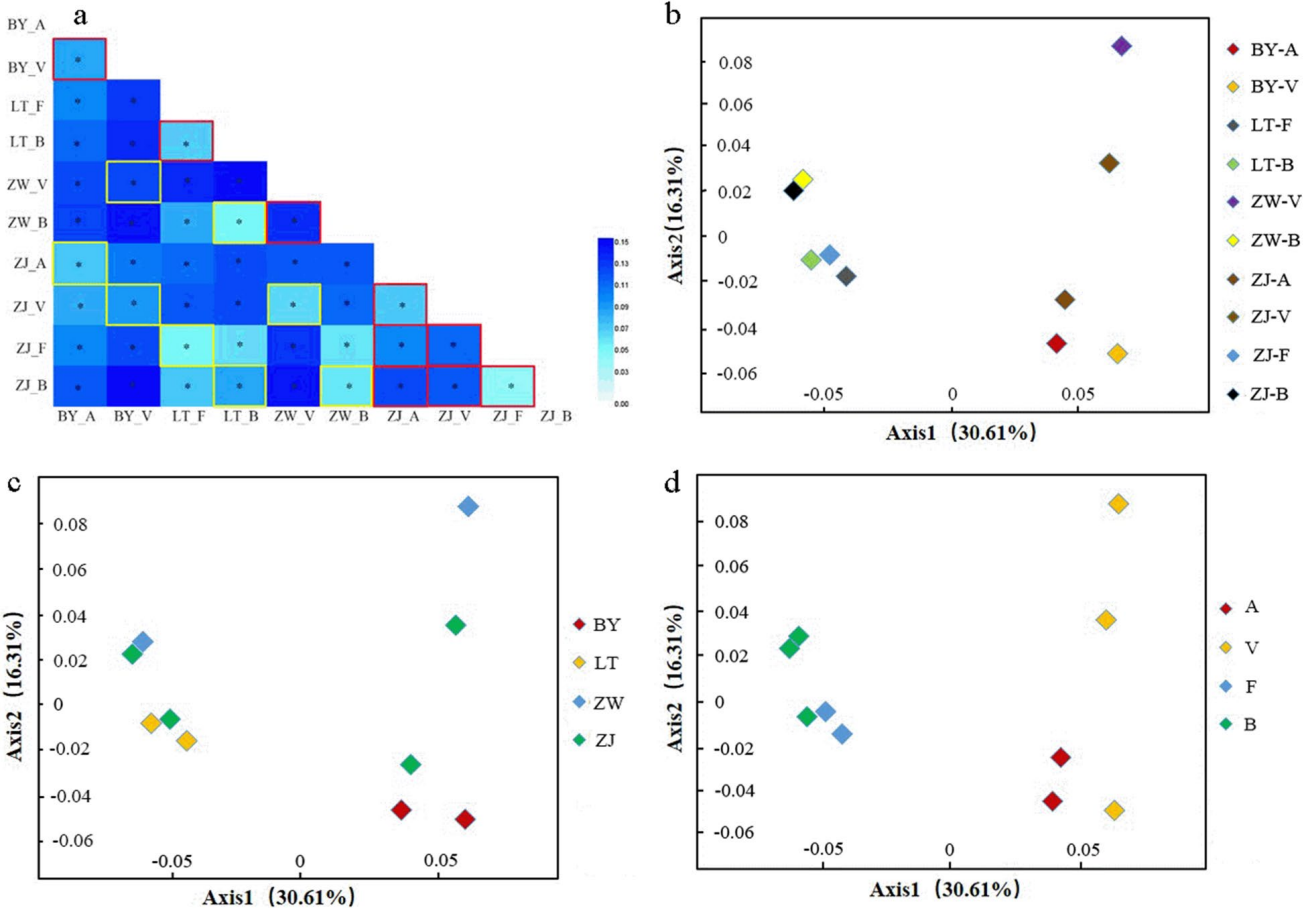
Pairwise *F*_ST_ in sympatric and allopatric populations of four oak species (**a**: the red box represents sympatric populations and the yellow box represents the same species). and principal coordinate analysis (PCoA) based on pairwise population *F*_ST_ values implemented in GENALEX 6.5 (**b**-**d**)

Null alleles have particular effect on the estimation of population differentiation, which was indicated by the almost equal values and similar 95% level CIs of global *F*_ST_ across all loci with (c*F*_ST_ = 0.085, 95% CI: 0.065—0.105) and without (*F*_ST_ = 0.101, 95% CI: 0.078–0.125) using the “exclusion null alleles” correction (Table 1). For each locus, c*F*_ST_ (range: 0.033 to 0.168) and standardized differentiation *G*′_ST_ (range: 0.033 to 0.190) were relatively low at the nominal level (5%). Thus, a weak genetic differentiation occurred among the four-oak species. We also compared the genetic differentiation between pairs of populations (Fig. 1a). In this study, we identified the same species as homologous and different species as heterogeneous. Population differentiation analysis revealed the following: allopatric homologous (mean *F*_ST_ = 0.064) < sympatric heterogeneous (*F*_ST_ = 0.071) < allopatric heterogeneous (*F*_ST_ = 0.084). The interspecific genetic differentiation levels were more significant than genetic differentiation among regions as observed using two-sided *P*-values (Table 3). The PCoA confirmed a strong genetic structure existed among the populations (Fig. 1b–d).

**Table 3:** Comparison of genetic statistics among the four *Quercus*. L groups

	Value of genetic statistics					Two-sided *P*-value			
	*A*_R_	*H*_O_	*H*_S_	*F*_ST_	group pair	*A*_R_	*H*_O_	*H*_S_	*F*_ST_
**Allopatric**					BY vs LT	0.642	0.154	0.390	0.708
Group BY	10.7	0.543	0.823	0.085	BY vs ZW	0.205	0.569	0.453	0.331
Group LT	11.2	0.662	0.847	0.067	BY vs ZJ	0.761	0.893	0.537	0.837
Group ZW	9.5	0.490	0.802	0.131	LT vs ZW	0.066	0.033*	0.072	0.152
Group ZJ	11.0	0.533	0.838	0.092	LT vs ZJ	0.794	0.057	0.750	0.518
					ZW vs ZJ	0.041*	0.573	0.099	0.261
**Heterogeneous**									
Group (AV)	10.3	0.497	0.817	0.090					
Group (FB)	11.0	0.607	0.842	0.062	AV vs FB	0.223	0.014*	0.139	0.078
Subgroup					A vs V	0.074	0.437	0.097	0.611
Group A	11.2	0.536	0.841	0.07	A vs F	0.892	0.231	0.523	0.609
Group V	9.7	0.471	0.801	0.089	A vs B	0.724	0.635	0.619	0.863
Group F	11.3	0.640	0.859	0.050	V vs F	0.040*	0.014*	0.007**	0.330
Group B	10.9	0.578	0.827	0.062	V vs B	0.127	0.121	0.210	0.286
					F vs B	0.657	0.447	0.211	0.769

*A*_R_ allelic richness with rarefaction to a common sample size of 10, *H*_O_ observed heterozygosity, *H*_*S*_ genetic diversity within populations, *F*_ST_ genetic differentiation index. *P < 0.05. **P < 0.01. Two-sided *P*-value was obtained after 10,000 permutations and taken as the proportion of random data sets giving larger statistics than the observed statistics

**Fig. 2:**
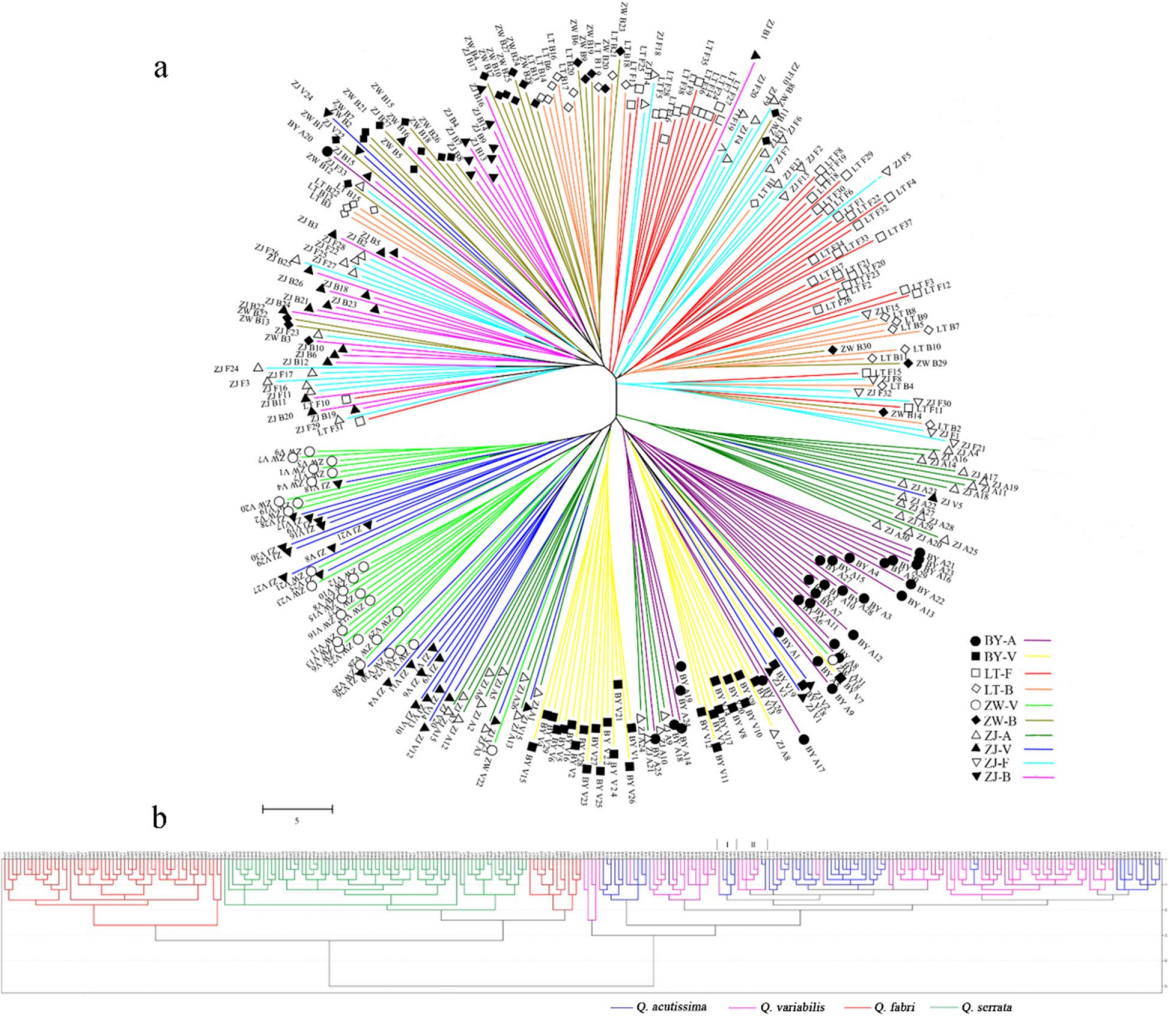
Molecular and phenotypic cluster analysis of 300 oak individuals. **a** NJ dendrogram showing genetic relationships implemented in MEGA 7.0 and **b** cluster analysis based on phenotypic traits variation

**Fig. 3:**
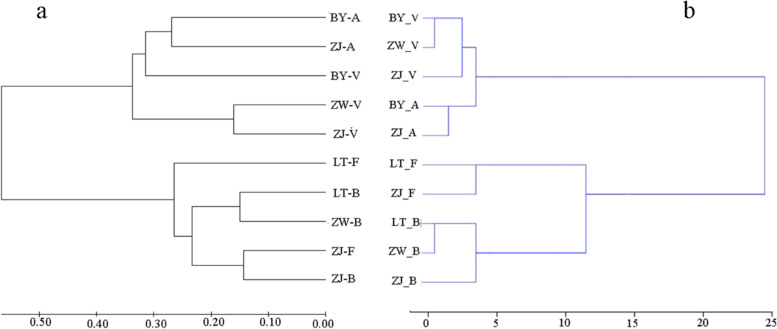
Molecular and phenotypic cluster analysis of 10 oak populations. **a** NJ cluster analysis implemented in MEGA 7.0 (Sudhir et al. 2016) based on the Nei’s unbiased genetic distances (Nei 1978). **b** Cluster analysis based on phenotypic traits variation

AMOVA results indicated that 1.04% (*P* = 0.003) of the genetic variation occurred among regions and 9.78% (*P* < 0.0001) among populations within regions, with the greatest genetic differentiation of 89.17% (*P* < 0.0001) being harbored within populations (Table 4). The hierarchical AMOVA revealed a significant genetic differentiation among species (*F*_CT_ = 0.04064, *P* < 0.001), with 7.2% (*P* < 0.0001) occurring among populations within species and the greatest genetic differentiation of 88.74% (*P* < 0.0001) being harbored within populations. The intraspecific gene flow of *Q. acutissima*, *Q. variabilis*, *Q. fabri*, and *Q. serrata* were estimated to be 3.31, 2.56, 4.72, and 3.8, respectively (Table 5). Simultaneously, the genetic variation among species was greater than among regions. In total, 300 samples were clustered into two branches based on Nei’s genetic distances using the NJ method (Fig. 2). *Q. acutissima* and *Q. variabilis* individuals were clustered into a large branch below the tree (group AV) and *Q. fabri* and *Q. serrata* (group FB) individuals were clustered into another large branch above the tree. The same species, along with other species, showed intraspecific differentiations and interspecies variations. Additionally, NJ graph showed considerable variation and somehow weak clustering of *Q. fabri* and *Q. serrata.* The clustering results of leaf variation were basically consistent with the molecular results. We clustered the four species and found that 14 individuals of *Q. fabri* and all of *Q. serrata* samples clustered into a single branch. These results indicated that the leaf traits were quite different from those of other *Q. fabri* individuals. There was a small deviation at the population-level clustering, which mainly occurred for the *Q. variabilis* population in Bayan, as well as for *Q. fabri* and *Q. serrata* populations in Liangting and Zijinshan (Fig. 3). This indicated that there was no great difference in morphology, but that the genetic structure had changed.

**Table 4:** Analysis of molecular variance (AMOVA) among the four *Quercus*. L groups

Source of variation	Degree of freedom	Sum of squares	Variance components	Percentage of variation	Fixation indices
***Allopatric***					
Among regions	3	197.815	0.0821	1.04	*F*_CT_ = 0.01044
Among populations within regions	6	315.516	0.76946	9.78	*F*_SC_ = 0.09887**
Within populations	590	4137.551	7.0128	89.17	*F*_ST_ = 0.10828**
***Heterogeneous***					
Among species	3	269.453	0.3212	4.06	*F*_CT_ = 0.04064*
Among populations within species	6	243.878	0.56879	7.2	*F*_SC_ = 0.07502**
Within populations	590	4137.551	7.0128	88.74	*F*_ST_ = 0.11262**

*F*_CT_, *F*_SC_ and *F*_ST_: genetic differences among regions (sub-regions), among populations within regions (sub-regions), and among all populations, respectively. *P < 0.00, **P < 0.0001. *P*-value was obtained through 10,000 permutations

**Table 5:** The gene flow (*N*_*m*_) among the 10 oak populations

Population^a^	BY-A	BY-V	LT-F	LT-B	ZW-V	ZW-B	ZJ-A	ZJ-V	ZJ-F	ZJ-B
BY-A	–									
BY-V	4.02	–								
LT-F	3.79	2.87	–							
LT-B	3.19	2.64	5.08	–						
ZW-V	2.95	2.82	2.65	2.37	–					
ZW-B	2.88	2.48	4.53	6.22	2.68	–				
ZJ-A	4.81	3.60	3.49	3.00	3.06	3.09	–			
ZJ-V	4.33	3.97	3.17	2.93	5.51	3.29	4.98	–		
ZJ-F	3.71	3.06	6.85	5.48	2.75	5.74	3.79	3.34	–	
ZJ-B	3.00	2.30	4.91	3.92	2.46	5.89	2.88	3.10	7.32	–

^a^see text for population code; *N*_*m*_ = estimate of gene flow from *G*_ST_, *N*_*m*_ = 0.5(1—*G*_ST_)/*G*_ST_

### Admixture analysis and hybrid identification

**Fig. 4:**
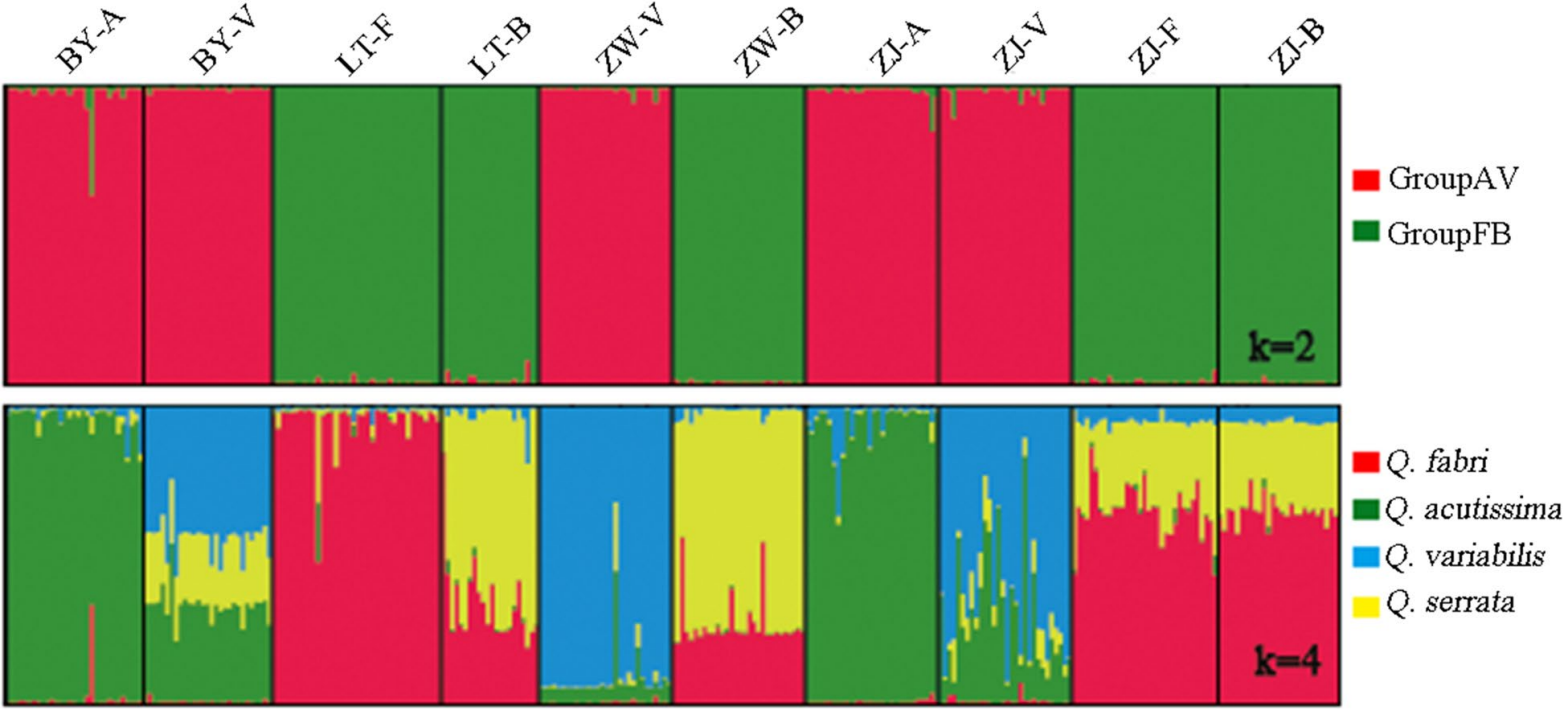
STRUCTURE clustering results for 300 oak species individuals from 10 populations (each color represents a genetic cluster)

Delta *K*, which is used to determine the best fit value of *K*, was computed using STRUCTURE HARVESTER for the given range, 1–9, and the highest value was obtained at *K* = 2 (Fig. 4). This was consistent with the taxonomic classifications of these four distinct species. In total, 98% of *Q. acutissima* and *Q. variabilis* individuals were assigned to cluster I (*Q*I), with an average proportion of membership (*Q*I = 0.98), and most *Q. fabri* and *Q. serrata* individuals (100%) were assigned to cluster II (*Q*II = 0.99). Only three remaining individuals (two *Q. acutissima* and one *Q. variabilis*) showed different degree of admixture with assignment probabilities of < 0.90. The 10 populations were divided into two large groups (Groups AV and FB). Using *K* = 4, the hybridization scenario among the four oak species was more complicated. *Q. fabri* individuals were assigned to cluster I (*Q*I = 0.80), *Q. acutissima* individuals were assigned to cluster II (*Q*II = 0.94), *Q. serrata* individuals were assigned to cluster III (*Q*III = 0.54), and *Q. variabilis* individuals were assigned to cluster IV (*Q*IV = 0.66) (Table S2).

We observed four types of hybridization, *Q. acutissima*—*Q. fabri*, *Q. acutissima*—*Q. variabilis*, *Q. fabri*-*Q. serrata*, and *Q. acutissima*—*Q. variabilis—Q. fabri* (Table 6). A total of 188 heterozygous individuals were observed (overall *H*r = 62.77%), including 67 F_1_ hybrids and 121 backcross individuals. Among the four sampled plots, the highest hybridization rate was found in Bayan plots (100%). Among the four-oak species, the hybridization rate was as follows: *Q. acutissima* (*H*r = 18.0%) < *Q. fabri* (*H*r = 50.7%) < *Q. variabilis* (*H*r = 74.2%) < *Q. serrata* (*H*r = 94.9%). Most hybridizations occurred in the same groups (AV or FB); however, there were also introgression individuals produced by cross breeding among different groups. Among them, individuals of *Q. variabilis* hybrids in Bayan were unique, having three types of ancestry (*Q. acutissima*—*Q. variabilis—Q. fabri*).

**Table 6:** Identification results of interspecies introgression between oak species

Population^a^	HT	NH (F_1_)	NH (backcross)	H_r_ (%)	N
BY-A	A-F	1	3	12.90	31
BY-V	A-V-B	29	0	100.00	29
LT-F	F-B	1	2	7.90	38
LT-B	B-F	8	13	95.50	22
ZW-V	A-V	5	3	27.00	30
ZW-B	F-B	1	26	90.00	30
ZJ-A	A-V	1	6	23.30	30
ZJ-V	V-A	10	19	96.70	30
ZJ-F	F-B	7	26	100.00	33
ZJ-B	B-F	4	23	100.00	27
Overall	-	67	121	62.77	300

^a^see text for population code; HT, hybrid type; NH, number of hybrids; H_r_, hybridization rate; N, sample size

Rate of hybridization per population: (NH_F1_ + NH_B_)/N_pop_; Rate of hybridization per species: (NH_F1_ + NH_B_)/N_species_; Overall hybridization rate: (NH_F1_ + NH_B_)/N_overall_

## Discussion

Introgression studies usually focus on large-scale pairs of individual species [29] and sympatric related species [30], but rarely species within distinct ecological niches and evolutionarily clades. Here, we examined the extent of introgression that existed among four oaks belonging to two different groups of sympatric and allopatric populations. Oak (*Quercus*) are notorious for hybridization with species extinction, and this may also play a critical role in adaptive evolution [31, 32]. Hybridization and introgression in oaks has been well documented, and adaptive introgression was important in evolution as revealed by genomics [11, 32–34]. Our study used nSSR markers to investigate the introgression of four oaks. We identified four types of hybrids: *Q. acutissima* × *Q. variabilis*, *Q. fabri* × *Q. serrata*, *Q. acutissima* × *Q. fabri*, and *Q. acutissima* × *Q. variabilis* × *Q. fabri*. Introgression occurred more readily in the *Quercus* group than in the *Cerris* group, and cross-group hybridization also existed; however, it was rare. This suggested that a low frequency of interspecific hybridization occurred with incomplete reproductive isolation. Reproductive isolation mechanisms contributed to the simultaneous coexistence of the four oaks.

### Genetic diversity and genetic variation of the studied four oak species

Introgression is a vital source of novel genetic variation and drives diversification during reticulate evolution [3]. Owing to the critical variation introduced by hybridization, and the hybrid constitution itself, the diversification rate shifts [2]. In this study, we found that the studied 10 oak populations, all had high levels of genetic diversity, with *Q. fabri* in the Liangting and Zijing sites having the highest value. Long-lived trees span broad geographical ranges through a combination of adaptive plasticity and adaptive differentiation, as well as the standing genetic variation that occurs in response to environmental changes [4]. A high genetic diversity provides adaptability that ensures the survival of the four oaks when facing extreme climatic events. In our study, the *F*_ST_ values of the 10 populations ranged from 0.05 to 0.15, which indicates moderate differentiation. Additionally, the order of of genetic differentiation among the 10 populations was as follows: allopatric homologous < sympatric heterogeneous < allopatric heterogeneous. *Q. acutissima* and *Q. variabilis* diverged 13.2 Ma, and the divergence time of *Q. fabri* and *Q. acutissima* was 12.33 Ma [35, 36]. During the quaternary ice period, the three species (*Q. acutissima*, *Q. variabilis*, and *Q. fabri*) showed different migration routes in response to climatic changes. In addition, the strong uplift of the Tibetan Plateau in China led to the intensification of the Asian monsoon climate, which may promote species differentiation [37–39]. The main cause of the genetic differentiation was the long-term evolutionary divergence of the four species, followed by adaptive differentiation caused by interspecific introgression [40].

By studying 17 pairs of polymorphic loci with different *F*_ST_ values, we speculated that gene flow occurred among these oaks. The limited gene flow prevented hybridization-induced genetic swamping, instead of providing more sources of genetic variation for heterozygotes. Additionally, we found that genetic variation among the four oaks mainly occurred within populations (88.74%), with only a small percentage occurring among populations (7.2%). Ramırez-Valiente et al. (2018) found that drought drove the evolution of genetic differences in functional traits among oaks [41]. The cluster analysis of leaf variation of the 300 individuals also showed large phenotypic variation. Allelic and associated phenotypic changes of species are strategies used to respond to the threat of global climatic changes during the adaptive evolutionary progress [42]. Although a small number of nuclear loci were used in this experiment to verify this hypothesis, we could not determine a phenotype associated with introgressed loci. A future genome-wide association analysis will further analyze the ingression regions and phenotypic associations.

### Gene flow and hybridization among the four-oak species

Compared with intraspecific gene flow dynamics, interspecific introgression is more complex owing to the interactions of multiple species. Gene flow levels were distinguished as high (*N*_*m*_ > 1), intermediate (0.25 < *N*_*m*_ < 0.99), and low (*N*_*m*_ < 0.25) by Govindaraju [43]. In our study, the intraspecific gene flows of *Q. acutissima*, *Q. variabilis*, *Q. fabri*, and *Q. serrata* were estimated to be 3.31, 2.56, 4.72, and 3.8, respectively. Compared with species in other families, such as *Picea asperata* (0.75) and *Liriodendron chinense* (1.028), the gene flow was stronger, being similar to those of other oak species, such as *Quercus aquifolioides* (3.749). Gene flow is an important attribute affecting species ability to spread its genes [44]. Most oak trees have larger pollen dispersal distances. The dispersal capacity in the genus *Quercus* is also related to many factors, such as tree height, vegetation density, and leaf area [45]. A small amount of gene flow enables all breeding populations to maintain similar alleles and thus high heterozygosity, so the NJ clustering graph showed weak differentiation between *Q.fabri* and *Q. serrata* groups while the STRUCTURE clustering revealed similar genetic separation. However, it may also be caused by variation within population, as stated above, the proportion of variation within population is very high (88.74%). We found recurrent interspecific gene flow among the four oaks. The STRUCTURE clustering revealed that most populations contained hybrids between two species. For example, the ZJ-A population showed F_1_ hybrids between *Q. acutissima* and *Q. variabilis*, while BY-A population showed hybridization between *Q. acutissima* and *Q. fabri*. Interestingly, a large number of individuals in the BY-V population appeared to be trihybrids. Additionally, STRUCTURE clustering showed that ZJ-F and ZJ-B populations did not form two strong groups and their hybrid individuals are relatively uniform, probably representing a mixed group. However, the phenotypic data indicated that these are two distinct species. Additionally, these two populations, BY-V and ZW-B, have more uniform hybrids, and we are not sure whether this is due to ancestral lineage or recent gene flow, and furthermore we did not rule out the existing possibility of shared variation. Because no suitable outgroups were utilized in the present study, the observed complex admixture patterns maybe are attributable to their ancestral contributions from other *Quercus* spp. Additional samples of related species are needed to elucidate this case. At the same time, the Bayan site appeared to be an especially active zone of introgression among these three oaks.

Many F_1_ progeny and some backcrosses were hybrids. In section *Cerris*, the most hybrids occurred among *Q. variabilis* backcrosses, while in section *Quercus*, the most hybrids occurred among *Q. serrata* backcrosses. This may have resulted from our sampling of individuals displaying more than one species traits, but we cannot rule out a bias toward introgressions between parental species. Such asymmetric introgression is common [46], as in the introgression from the coastal oak species *Quercus dentata* to an ecotype of the inland oak species *Quercus mongolica* var*. crispula*, which has colonized the coastal environment in northern Japan [34]. The environment in which hybrid individuals are grown is an important cause of asymmetric introgression [47–49]. Oaks are wind-pollinated, and gene flow mainly occurs through pollen [50]. Therefore, differentiation in seed and pollen dispersal between hybrids and parental species are crucial. Future sampling should target a broader range of individuals in unique environments, like the edge of a hybrid zone, to determine whether their asymmetric introgression is occurring at the genome level. In addition, the reproductive isolation patterns between the hybrid individuals and their parents would be experimentally verified.

### Implications for forest management

Natural hybridization is a common phenomenon in plants, occurring in 25% of existing species [51, 52]. Fertile hybrids backcross with their parents, leading to introgression. Introgression is an evolutionary creative force that introduces new, possibly adaptive alleles into a population [53, 54]; however, excessive gene flow may result in genetic swamping and species extinction [55]. For conservationists, whether to prevent or encourage interspecific gene flow is a difficult question. Oaks are “notorious” for their widespread hybridization and are exceptional forest trees with high levels of genetic diversity. Oaks tend to hybridize, and introgression may influence community structure and increasing genetic diversity [6]. In the face of climatic changes, interspecific gene flow transfers adaptive alleles to avoid extinction [56, 57]. The studied four oak species (*Q. acutissima*, *Q. variabilis*, *Q. fabri*, and *Q. serrata*) are all widely distributed in China. A palaeo distributive simulation showed that *Q. acutissima*, *Q. variabilis*, and *Q. fabri* tended toward expansion and contraction in glacial and interglacial periods, respectively [37–39]. Adaptive evolution in response to changing environment allows an organism to avoid extinction. Such adaptive evolution is related to the adaptive introgression of the species. In our study, hybridization and introgression existed among the studied four oak species. Although we did not confirm whether this introgression was adaptive, other experiments have confirmed this view [53, 54]. In future studies, we will use genome-wide data to correlate phenotypic data with fitness to explore the adaptive introgression of *Quercus*. To increase the efficacy of conservation strategies, conservation management should consider evolutionary theory. Thus, managers should consider whether hybrids co-exist with the parental species when formulating conservation strategies because natural hybrids have conservation value. Natural hybridization zones are good sites for studying species adaptive evolution using newly developed genomics techniques.

## Conclusions

The long-term evolutionary divergence of the studied four oaks, and the resulting interspecific introgression, led to their high levels of genetic diversity and moderate differentiation. The genetic variation among the four oaks mainly occurred within populations, with only a small percentage occurring among populations. Four types of hybrids (*Q. acutissima* × *Q. variabilis*, *Q. fabri* × *Q. serrata*, *Q. acutissima* × *Q. fabri*, and *Q. acutissima* × *Q. variabilis* × *Q. fabri*) were determined, accompanied by asymmetric introgression. We concluded that interspecific hybridization is commonly observed within the section and that section *Quercus* has a high tendency to hybridize. In future work, we hope to use genomic data to study the dynamic nature of gene flow and adaptive introgression in these hybrid populations.

## Methods

### Sampling and study sites

**Fig. 5:**
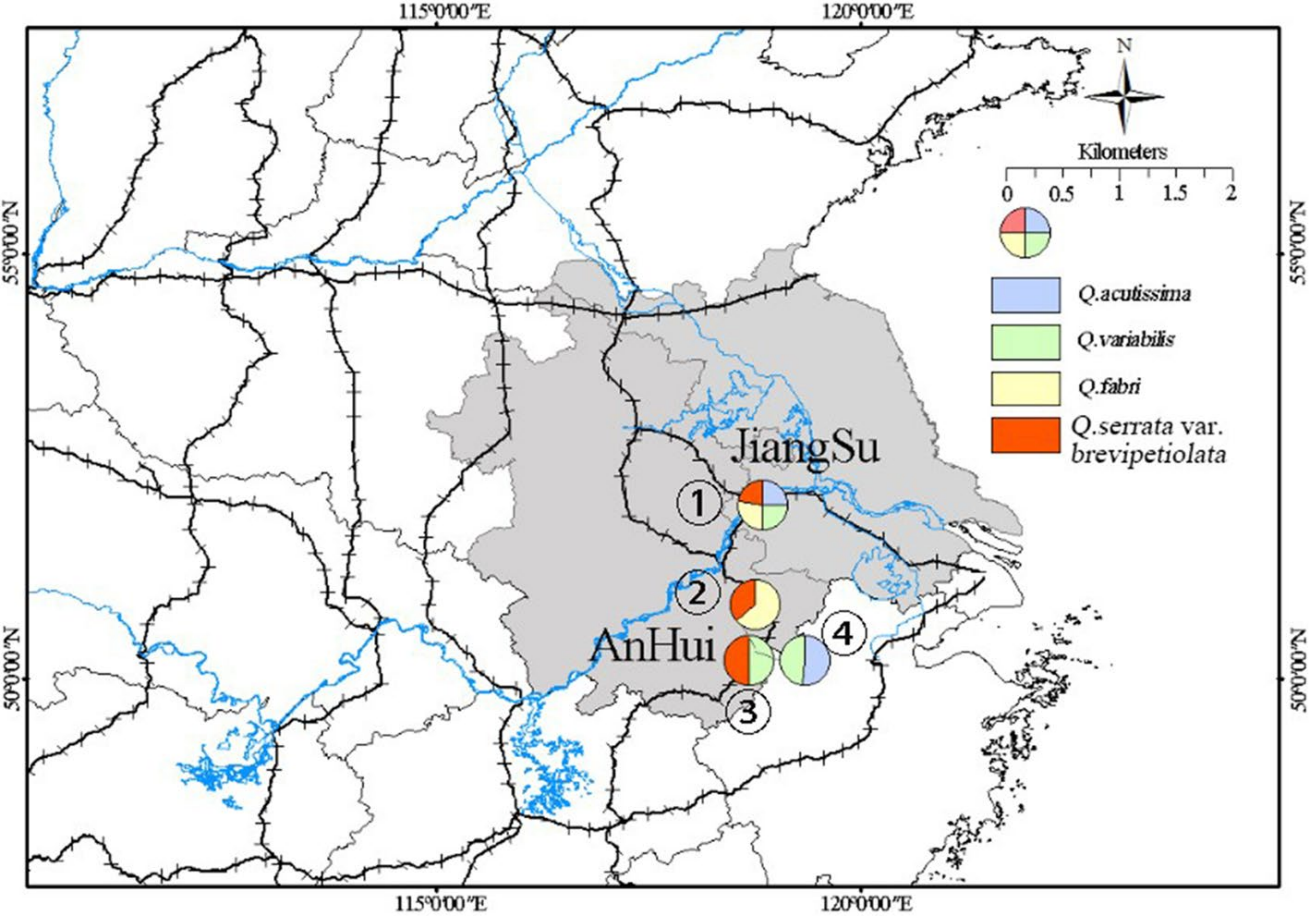
Geographic distribution of the sampled four oak plots. Pie charts with different color represent different species (*Q. acutissima*: blue; *Q. variabilis*: green; *Q. fabri*: yellow; *Q. serrata*: red). Pie area is proportional to the sample size

In this study, we used the new classification of *Quercus* proposed by Denk et al. (2017) in which *Quercus* is divided into eight sections: *Cyclobalanopsis*, *Cerris*, *Ilex*, *Lobatae*, *Quercus*, *Ponticae*, *Protobalanus*, and *Virentes* [27]. We selected four species in section *Quercus*, two wild populations of *Q. acutissima*, three of *Q. variabilis*, two of *Q. fabri*, and three of *Q. serrata*, for genetic analyses, and they were sampled randomly within four plots distributed in Jiangsu Province (Plot Zijing) and Anhui Province (Plots Bayan, Liangting, and Zhongwu) (Fig. 5). Within each locality, selected individuals were at least 30 m apart to avoid sampling the same plant. A total of 300 adult individuals were sampled, and their geographic coordinates and altitudes were recorded using a global positioning system (Table S3). From each individual plant, three to five leaves were collected, quickly dried in silica gel, and stored at room temperature for molecular experiment. At the same time, we randomly collected 10–15 leaves for morphological analysis. A single branch was collected for identification by Y.F. and then preserved in Nanjing Forestry University herbarium.

### Measurement of leaf morphological traits

Nine leaf morphological attributes were measured using 10 mature leaves from each of the 300 trees (Table 4 S). The observed variables were scored as indicated in Table 4S. The dimensional characteristics were measured with a ruler. The epidermal hairs were observed under a microscope at 40 × magnification.

### DNA extraction and microsatellite genotyping

Total genomic DNA was extracted from 30–40 mg dried leaves per individual using a Plant Genomic DNA Kit (Tiangen, China). The quality and concentration of the genomic DNA were evaluated using a One-drop spectrophotometer (OD–1000, Shanghai Cytoeasy Biotech Co., Ltd., China) and electrophoresis was conducted in 1% agarose gels. DNA samples were diluted to 20 ng/μL and stored at -20 °C.

All DNA samples were genotyped at 17 nuclear microsatellite loci (nSSRs) (QM58TGT [58]; quru-GA-0I01, quru-GA-0M05, quru-GA-0M07, Quru-GA-Oi21, Quru-GA-1H14, Quru-GA-1i15 [59, 60]; MSQ16 [61]; ssrQpZAG1/5, ssrQpZAG15, ssrQpZAG36 [62]; ssrQrZAG 7, ssrQrZAG 31, ssrQrZAG 74, ssrQrZAG 87, ssrQrZAG 96, and ssrQrZAG 112 [63]), using primers developed for oak tree species (Table S5). PCR reactions were performed in an Eppendorf thermal cycler (Eppendorf, Hamburg, Germany) using the following parameters: initial denaturation at 94 °C for 4 min, 30 cycles of denaturation at 94 °C for 45 s, 45 s of annealing at a primer specific temperature, and extension at 72 °C for 45 s, followed by a final extension of 8 min at 72 °C. The PCR mixtures had total volumes of 20 μL, containing 2 × Taq PCR MasterMix (Tiangen), 10 μM of each primer, 20–40 ng of template DNA, and ddH_2_O. PCR products were separated on an ABI3730xl automated Genetic Analyzer using ROX-500 as an internal standard (Applied Biosystems, USA). Allele sizes were determined manually using Genemarker version 2.2.0 (Applied Biosystems).

### Statistical analyses

We used the dimensional characteristics, transformed variables, counted variables, and observed variables described in the literature to detect the intraspecific variation. The morphological characteristics data was analyzed using SPSS 19.0 (SPSS, Inc., Chicago, IL, USA). Using the Euclidean distances, nine phenotypic characteristics were clustered at the individual and population level. The obtained population clustering graphs and individual clustering diagrams were used along with the molecular results in a comparative analysis.

For the SSR data, we used INEST, version 2.2 [64] to detect null allele frequency at each locus simultaneously in each population using the individual inbreeding model, which includes three parameters, null alleles (*n*), inbreeding coefficients (*f*), and genotyping failures (*b*). The number of Markov Chain Monte Carlo iterations and burn-in were set at 500,000 and 50,000 cycles, respectively. Thinning maintained every 50^th^ update. To conduct a Bayesian model comparison, we performed the analysis using the model with null alleles (*nfb*) and without null alleles (*fb*) to evaluate the significance of null alleles within each population. We set *nfb* significance using low deviance information criterion (DIC). For this method, the thresholds were set to a maximum of less than 0.2 and a mean of less than 0.1, which resulted in the final selection of all 17 loci for subsequent analyses. Linkage disequilibrium for all the locus pairs in each population and significant deviations from Hardy–Weinberg equilibrium were determined using Genepop version 4.6.9 [65].

Genetic diversity statistics of each population and each locus were estimated using POPGENE, version 1.32 [66]. The statistics included number of alleles (*N*), observed (*H*_O_) and expected (*H*_E_) heterozygosity and effective number of alleles (*N*_E_). Polymorphic information content (*PIC*) was estimated in Cervus 2.0 [67], while allelic richness (*A*_R_), genetic diversity within populations (*H*_S_), and inbreeding coefficient (*F*_IS_) were estimated using FSTAT version 2.9.3.2 [68]. The corrected inbreeding coefficient (*F*_IS_′) for each population was also evaluated using INEST version 2.2 [64] with the full model (*nfb*). The significance of *F*_IS_′ was assessed by comparing DIC values of inbreeding and without inbreeding models.

We estimated global *F*_ST_ and c*F*_ST_ [69] both with and without the “exclusion null allele” correction per loci using FreeNA [70]. The 95% level confidence intervals (CIs) of both global *F*_ST_ and c*F*_ST_ across loci were obtained through bootstrap resampling in the same program. The *F*_ST_ of each pair of populations was estimated in GENALEX 6.5 [71] and visualized in the form of a heatmap using Heml 1.0.33 [72]. Gene flow was indirectly estimated using Wright’s (1951) formula: *Nm* = 1—*F*_ST_/4 *F*_ST_. Additionally, we performed a principal coordinate analysis based on pairwise population *F*_ST_ values using GENALEX 6.5 [71]. We tested the significance of differences in *A*_R_, *H*_O_, *H*_S_, and *F*_ST_ among geographic regions and among species using FSTAT, version 2.9.3.2 [68]. The two-sided *P*-values were obtained after 10,000 permutations. To examine the genetic variances of allopatric and heterogeneous types we conducted analysis of molecular variance (AMOVA) using *F*-statistics using ARLEQUIN, version 3.5 [73]. Significances of fixation indices (*F*_CT_, *F*_SC_, and *F*_ST_) were tested with 10,000 permutations.

STRUCTURE software [74] was used to infer population structure. To identify the number of populations (*K*) capturing the data major structure, a burn-in period of 500,000 Markov Chain Monte Carlo iterations was used, with a 500,000-run length. In total, 20 independent runs were performed for each simulated *K* value, ranging from 1 to 9. All the iterations were run with the admixture model, which assumes that individuals may have mixed ancestry, because of the likelihood of inter-population and interspecific crossing among the four oaks. The optimal *K* value was identified from the maximum value of Δ*K* [75] as implemented using STRUCTURE HARVESTER 0.6.93 [76]. Clusters of 20 runs were permuted using CLUMPP, version 1.1.2 [77], and DISTRUCT 1.1 [78] was employed to envisage the STRUCTURE results after processing with CLUMPP. On the basis of the binary matrices, Nei’s unbiased genetic distance matrix was calculated using GENALEX 6.5 [71], and was employed to construct a dendrogram using MEGA 7.0 [79] with the neighbor-joining (NJ) clustering method [80].

The admixture coefficient (q-value) generated from STRUCTURE was used to classify individuals into purebreds and hybrids, with a threshold q-value of = 0.1, where samples with q-values < 0.1 or > 0.9 were classified as purebreds, and those with 0.1 < q-values < 0.9 as hybrids, including the F_1_ generation and backcrosses [81, 82]. F_1_ hybrids have q-values = 0.5, but the coefficients of backcrosses are biased toward one of the parental species and produce q-values between 0.5 and 0.9 [81]. Taking errors into consideration, individuals with 0.6 < q-values < 0.9 were recognized as backcrosses.

## Supplementary Information

Additional file 1:**Table S1.** Deviance information criterion values for nfb, nb, and fb models in 10 populations obtained by INEST. **Table S2.** Probability of membership to each genetic cluster in each population of four oaks when K = 2 and 4. **Table S3.** Locations of 10 populations of four oak species. **Table 4S.**. Leaf macromorphological features examined. **Table S5.** SSR primer sets, number and size of alleles amplified in four oak species study.

## Data Availability

The SSRs genotyping data from this study have been deposited in GitHub (https://github.com/Lixuan221/SSR-Database).

## Abbreviations

SSRs: Simple sequence repeats
AFLPs: Amplified fragment length polymorphisms
RAD-seq: Restriction-site associated DNA sequencing
SNPs: Single nucleotide polymorphism
*N*_A_: Number of alleles
*N*_E_: Effective number of alleles
*A*_R_: Allelic richness
*H*_O_: Observed heterozygosity averaged across loci
*H*_E_: Expected heterozygosity averaged across loci
*H*_S_: Genetic diversity within populations averaged across loci
*PIC*: Polymorphism information content
*F*_ST_: Genetic differentiation index
c*F*_ST_: Corrected genetic differentiation index using the “exclusion null alleles” (ENA) method
*G'*_ST_: Standardized genetic differentiation index
*T*_A_: Annealing temperature
null: Null allele frequency averaged across populations
DIC: Deviance information criterion value
*n*: Model with null alleles
*f*: Inbreeding coefficients
*b*: Genotyping failures
