# Supplementary material for: Hybridization and introgression in sympatric and allopatric populations of four oak species
Source: BMC Plant Biol. 2021 Jun 9;21:266. doi: 10.1186/s12870-021-03007-4 (PMC8188795; doi:10.1186/s12870-021-03007-4)
Supplement: Supplementary file 1 — Additional file 1:Table S1. Deviance information criterion values for nfb, nb, and fb models in 10 populations obtained by INEST. Table S2. Probability of membership to each genetic cluster in each population of four oaks when K = 2 and 4. Table S3. Locations of 10 populations of four oak species. Table 4S.. Leaf macromorphological features examined. Table S5. SSR primer sets, number and size of alleles amplified in four oak species study. [file 12870_2021_3007_MOESM1_ESM.docx]

**Additional Files:**

**Table 1S** Deviance information criterion values for nfb, nb, and fb models in 10 populations obtained by INEST

**Table 2S** Probability of membership to each genetic cluster in each population of four oaks when K = 2 and 4

**Table 3S** Locations of 10 populations of four oak species

**Table 4S** Leaf macromorphological features examined

**Table 5S** SSR primer sets, number and size of alleles amplified in four oak species study

Table 1S Deviance information criterion values for *nfb*, *nb*, and *fb* models in 10 populations obtained by INEST

| Population code | DIC*nfb* | DIC*nb* | DIC*fb* | DIC*nfb*-DIC*nb* | DIC*nfb*-DIC*fb* |
| --- | --- | --- | --- | --- | --- |
| BY-A | 4123.33 | 4118.30 | 4142 | 5.0 | -18.70 |
| BY-V | 3305.49 | 3298.98 | 3393 | 6.51 | -87.51 |
| LT-F | 5021.55 | 5035.59 | 5099 | -14.04 | -77.45 |
| LT-B | 2875.49 | 2872.55 | 2887 | 2.94 | -11.51 |
| ZW-V | 3256.52 | 3252.18 | 3292 | 4.34 | -35.48 |
| ZW-B | 3641.31 | 3644.41 | 3675 | -3.10 | -33.69 |
| ZJ-A | 3703.91 | 3698.54 | 3716 | 5.37 | -12.09 |
| ZJ-V | 3638.28 | 3632.72 | 3659 | 5.56 | -20.72 |
| ZJ-F | 4451.18 | 4446.25 | 4465 | 4.93 | -13.82 |
| ZJ-B | 3480.94 | 3472.93 | 3508 | 8.01 | -27.06 |

DIC, deviance information criterion value; *nfb*, model with null alleles (*n*), inbreeding coefficients (*f*) and genotyping failures (*b*); *nb*, model with null alleles (*n*) and genotyping failures (*b*); *fb*, model with inbreeding coefficients (*f*) and genotyping failures (*b*).

Table 2S Probability of membership to each genetic cluster in each population of four oaks when *K* = 2 and 4

| Pop | group | *q*_AV_ (%) | *q*_FB_ (%) | *q*_I_ (%) | *q*_II_ (%) | *q*_III_ (%) | *q*_IV_ (%) |
| --- | --- | --- | --- | --- | --- | --- | --- |
| BY-A | AV | 97.8 | 2.2 | 1.74 | 94.42 | 1.62 | 2.22 |
| ZJ-A | AV | 98.7 | 1.3 | 0.67 | 93.59 | 1 | 4.74 |
| BY-V | AV | 99.5 | 0.5 | 0.59 | 32.67 | 22.7 | 44.05 |
| ZW-V | AV | 99.3 | 0.7 | 0.48 | 7.1 | 1.78 | 90.64 |
| ZJ-V | AV | 98.8 | 1.2 | 0.96 | 28.09 | 6.17 | 64.78 |
| LT-F | FB | 0.5 | 99.5 | 95.35 | 1.03 | 2.8 | 0.81 |
| ZJ-F | FB | 0.8 | 99.2 | 65.1 | 1.04 | 29.1 | 4.76 |
| LT-B | FB | 1.2 | 98.8 | 33.72 | 0.95 | 63.08 | 2.25 |
| ZW-B | FB | 0.6 | 99.4 | 26.67 | 0.47 | 71.4 | 1.46 |
| ZJ-B | FB | 0.4 | 99.6 | 64.83 | 0.6 | 29.68 | 4.9 |

Table 3S Locations of the oak 10 populations representing four species

| No. | Population code | Species | Simple size | Location |
| --- | --- | --- | --- | --- |
| 1 | BY-A | *Q. acutissima* | 31 | Bayantang, Ningguo, Anui, China |
| 2 | BY-V | *Q. variabilis* | 29 | Bayantang, Ningguo, Anui, China |
| 3 | LT-F | *Q. fabri* | 38 | Liangtinggoukou, Ningguo, Anui,China |
| 4 | LT-B | *Q. serrata* | 22 | Liangtinggoukou, Ningguo, Anui, China |
| 5 | ZW-V | *Q. variabilis* | 30 | Zhongwushan, Jixi town Anhui, China |
| 6 | ZW-B | *Q. serrata* | 30 | Zhongwushan, Jixi, Anhui, China |
| 7 | ZJ-A | *Q. acutissima* | 30 | Zijin mountain, Nanjing, Jiangsu, China |
| 8 | ZJ-V | *Q. variabilis* | 30 | Zijin mountain, Nanjing, Jiangsu, China |
| 9 | ZJ-F | *Q. fabri* | 33 | Zijin mountain, Nanjing, Jiangsu, China |
| 10 | ZJ-B | *Q. serrata* | 27 | Zijin mountain, Nanjing, Jiangsu, China |

Table 4S Leaf macromorphological attributes examined

| ID | Abbreviation | Description | Character code |
| --- | --- | --- | --- |
|  | dimensional characters |  |  |
| 1 | LL | Length of lamina |  |
| 2 | PL | Petiole length |  |
| 3 | MW | Middle width of lamina |  |
|  | transformed variables |  |  |
| 4 | LL/PL | Length of lamina/ Length of petiole |  |
|  | counted variables |  |  |
| 5 | NV | Number of intercalary veins |  |
|  | observed variables |  |  |
| 6 | SLB | Shape of lamina base | Wedge① Round or heart-shaped② Ear shape③ |
| 7 | SLA | Shape of lobe apex | Sharpening① Blunt or short acuminate② |
| 8 | SLM | Shape of lamina margin | Coarse or undulate teeth① Awn shape② glands③ |
| 9 | EH | Epidermic hairs | Cilia or glabrous① Stellate hairs② |

Table 5S SSR primer sets, number and size of alleles amplified in four oak species study

| locus | Repeat unit of cloned allele | Primer sequences (5'-3') | Allele size / bp | Reference | *T*_A_ (°C) |
| --- | --- | --- | --- | --- | --- |
| QM58TGT | (CAA)11 | GGTCAGTGTATTTTGTTGGT  AAATGTATTTTGCTTGCTCA | 212 | a | 52 |
| quru-GA-0I01 | (GA)16 | GCGCTATCAAGTAAGTGCTTAAC  ACGCCATCCCTATAACACA | 200 | b | 55 |
| quru-GA-0M05 | (GA)20 | CTACAAGTTACATGCCCAATCA  CTTTGCGCAGGTCCATTAC | 219 | b | 53 |
| quru-GA-0M07 | (GA)19 | TTTAGCATCACATTTCCGTT  TTTTGTGTCATCCGGTATTA | 209 | b | 45 |
| Quru-GA-Oi21 | (GA)16 | ATATGGTCCCGATTAATTC  GGGCAACATTCAAATGTATCTA |  | b | 50 |
| Quru-GA-1H14 | (GA)22 | GCTTGGGCTTGTTCCTACT  CAACACTTCTCATGGATTAGAGA |  | b | 58 |
| Quru-GA-1i15 | (GA)23 | CAGCCTCATCGATTACCCCAAAC  GGTCGCTGAGGGGGAAAG |  | b | 50 |
| MSQ16 | -- | GGAACAACTAGAGAGAAC  TTGCCTATCCTGCCCCGTAT |  | c | 52 |
| ssrQpZAG1/5 | (GT)5(GA)9 | GCTTGAGAGTTGAGATTTGT  GCAACACCCTTTAACTACCA |  | d | 58 |
| ssrQpZAG15 | (AG)23 | CGATTTGATAATGACACTATGG  CATCGACTCATTGTTAAGCAC |  | d | 52 |
| ssrQpZAG36 | (AG)19 | GATCAAAATTTGGAATATTAAGAGAG  ACTGTGGTGGTGAGTCTAACATGTAG |  | d | 57 |
| ssrQrZAG 7 | (TC)17 | CAACTTGGTGTTCGGATCAA  GTGCATTTCTTTTATAGCATTCAC | 150 | e | 51 |
| ssrQrZAG 31 | (GA)31 | CTTAGTTTGGTTGGGAAGAT  GCAACCAAACAAATGAAAT | 190 | e | 51 |
| ssrQrZAG 74 | (GA)23 | TTTATGTAGTGAGTAGTGGGGGTC  GCTATCATCCATCCCCCAACA | 124 | e | 55 |
| ssrQrZAG 87 | (TC)20 | TCCCACCACTTTGGTCTCTCA  GTTGTCAGCAGTGGGATGGGTA | 120 | e | 56 |
| ssrQrZAG 96 | (TC)20 | CCCAGTCACATCCACTACTGTCC  GGTTGGGAAAAGGAGATCAGA | 172 | e | 59 |
| ssrQrZAG 112 | (GA)32 | TTCTTGCTTTGGTGCGCG  GTGGTCAGAGACTCGGTAAGTATTC | 127 | e | 45 |

a: *C. myrsinifolia* (Isagi and Suhandono; 1997) b: *Q. rubra* (Aldrich *et al*., 2002; 2003); c: *Q. macrocarpa* (xuxiaolin et al, 2004; http://www.ncbi.nlm.nih.gov/probe/ 6103142); d: *Q. petraea*（Steinkellner *et al.*, 1997; e: *Q. robur* (Kampfer *et al*.1998)
